# Averaging rotational landmarks during total knee arthroplasty reduces component malrotation caused by femoral asymmetry

**DOI:** 10.1186/s13018-017-0575-2

**Published:** 2017-05-12

**Authors:** Tat Woon Chao, Liam Geraghty, Pandelis Dimitriou, Simon Talbot

**Affiliations:** Department of Orthopaedics, Western Health, 1/210 Burgundy Street, Heidelberg, Victoria 3084 Australia

**Keywords:** Knee, Arthroplasty, Malrotation, Prosthesis, Femoral rotation, Whiteside’s line, Knee replacement, Trochlear, Asymmetry

## Abstract

**Background:**

Femoral component malrotation is a common cause of patient dissatisfaction after total knee arthroplasty. The sulcus line (SL) is more accurate than Whiteside’s line as it corrects for variation in the coronal orientation of the groove. The hypothesis is that averaging the SL and posterior condylar axis (PCA) will reduce femoral malrotation.

**Methods:**

The component was inserted at a position between the SL and PCA in 91 patients. An intraoperative photograph was taken showing the landmarks. These were compared to the component position achieved relative to the surgical epicondylar axis (SEA) on a postoperative CT scan. The component position was compared to the position achieved using the individual landmarks.

**Results:**

Relative to the SEA, the final component position was 0.6° (SD 1.4°, range −3.8° to +4.0°), the coronally corrected SL position was −0.7° (SD 2.3°, −5.5° to +4.6°), the PCA position was 0.9° (SD 1.9°, −6.1° to +5.0°). Averaging the landmarks significantly decreased the variance of the component position compared to using the SL and PCA individually. The number of outliers (>3° from SEA) was also significantly less (*p* < 0.05) for the average position (2/84) when each was compared to the SL (16/84) and PCA (14/84) individually. In 21/84 (25%) of cases, there was more than 4° of divergence between the SL and PCA.

**Conclusions:**

Averaging the SL and the PCA decreases femoral component malrotation. Femora are frequently asymmetrical in the axial plane. Referencing posterior condyles alone to set rotation is likely to cause high rates of patellofemoral malalignment.

## Background

Femoral component rotation in total knee arthroplasty (TKA) continues to be a contentious issue. Femoral component malrotation is associated with patella maltracking [1–7], increased patella shear forces [8], flexion instability [9, 10], and soft tissue imbalance. There are several competing theories and techniques described which can be broadly separated into measured resection techniques based on anatomical landmarks and gap-balancing techniques based on ligament tension. The measured resection group can be further divided depending on the particular landmarks referenced. These include the anatomical epicondylar axis (AEA), the surgical epicondylar axis (SEA), the posterior condylar axis (PCA), kinematic alignment (KA) based on the posterior condylar line (PCL), the anteroposterior axis (APA, also known as Whiteside’s line) and, more recently, the sulcus line of the trochlear groove (SL).

These landmarks have been extensively studied to determine how they relate to each other and to the flexion-extension axis (FEA) of the knee. The SEA has been recommended as a closer approximation of the FEA of the knee than the AEA [11–15]. However, both landmarks are difficult to reliably isolate intraoperatively [16, 17].

Referencing the posterior condyles during either mechanical alignment or KA techniques relies on the assumption that the flexion-extension axis of the posterior condyles has a consistent relationship with the axis of rotation of the patella and trochlear groove. There is some research to suggest that on average, the axes are close to parallel [18, 19]. However, the relationship is not consistent, with a wide range of variation [20–24].

A new technique for measuring the rotational alignment of the trochlear groove has recently been developed [25, 26]. The APA has previously been the only technique used to assess the rotation of the trochlear. It has been shown to be unreliable due to parallax error [26]. The sulcus line (SL) technique considers the three-dimensional shape of the trochlear groove. It removes the parallax error by measuring the rotational alignment of the groove after reorienting the femur to look directly along the coronal alignment of the trochlear groove. This coronal direction varies widely between individuals [18, 26]. A simple instrument (Sulcus Line Trochlear Alignment Guide (STAG), (Enztec Ltd, Christchurch, New Zealand) has been developed to allow the SL to be used intraoperatively. The prediction from the previous three-dimensional computed tomography (3DCT) and cadaver studies [25, 26] is that averaging the SL and PCA would decrease the rate of femoral component malrotation.

The aims of this study were to (i) determine the clinical accuracy of the SL and STAG technique, (ii) assess the benefit obtained by averaging the SL and PCA, and (iii) characterise the relationships between the SL, SEA and posterior condyles.

## Methods

Approval to conduct the study was gained from the local Human Research Ethics Committee.

A prospective study of a consecutive series of 91 TKAs was conducted. There were no preoperative exclusion criteria. All operations were performed by one surgeon using a standardised technique. Conventional instruments were used. The distal femoral cut was produced at 6° from the anatomical axis of the femur using an intramedullary rod. The tibial cut was produced perpendicular to the long axis of the tibia using an extramedullary jig.

The femoral rotation was determined by averaging the SL and the PCA. The SL was drawn on the distal femur using diathermy after dislocation of the patella and before any bony cuts were made. This was done by carefully palpating the deepest points of the trochlear groove starting at the intercondylar notch and leading anteriorly. The most proximal section of the trochlear groove was not incorporated into the SL as it has been shown to be prone to excessive variability due to arthritic damage, osteophyte formation and anatomical variation [27, 28]. Multiple diathermy marks were made along the groove and then connected to produce a continuous curved line. A drill was then used to open the intramedullary canal in the centre point of the knee.

**Fig. 1:**
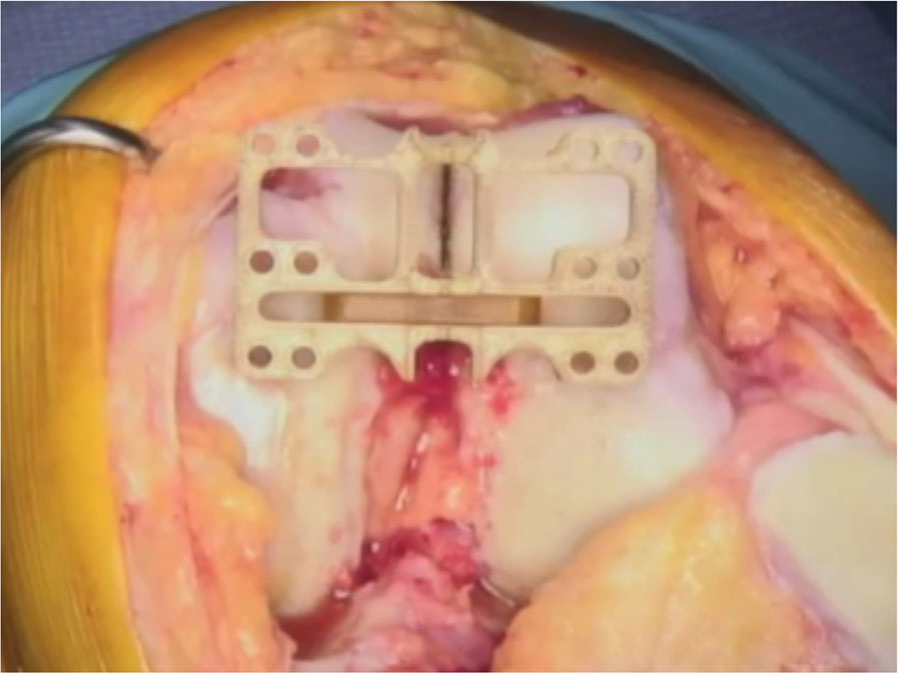
STAG device (axial view)

**Fig. 2:**
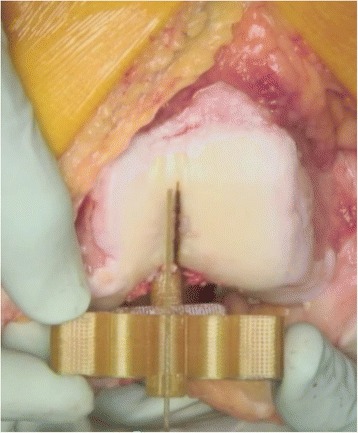
STAG device (coronal view)

The STAG intramedullary rod was inserted and the STAG block placed over the rod. The block was then orientated to match both the rotational (Fig. 1) and the coronal (Fig. 2) alignment of the SL. This was checked with an alignment wing in both planes. Care was taken to ensure that the block was perpendicular to the coronal alignment of the SL as viewed on the anterior surface of the femur. This usually left the block sitting off either the medial or lateral femoral condyle. Two smooth 3.2-mm pins were then drilled through parallel holes in the alignment block. The pins, block, and IM rod were then completely removed. The distal femoral cut was then produced using the standard technique described above without any reference to the SL or STAG device. After the distal femoral cut was performed, the holes made from the pin tracks from the STAG pin-holes were then identified on the cut surface.

The PCA was determined with the use of a standard sizing guide. Paddles were placed under the posterior condyles, and an additional 3° of external rotation was added to compensate for the average proximal tibia joint line obliquity. The PCA is defined as 3° of external rotation to the posterior condylar line (PCL). A 3.2-mm drill was used to produce two holes on the distal cut surface which match the rotation of the PCA. In order to report reproducible technique which does not rely on surgeon experience and to allow the calculation of an accurate PCL, no adjustment was made for posterior condylar bone loss.

**Fig. 3:**
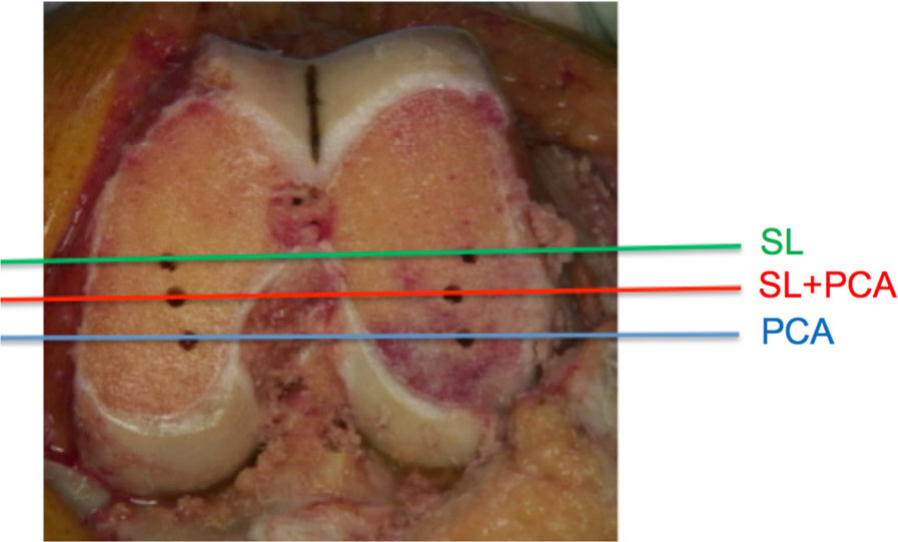
Pin-holes on distal femur

A sizing guide was used which allowed variation in the rotation from the posterior condyles in 1° increments between 0° and 6° of external rotation. The rotation of the SL (STAG pin-holes) and the PCA (PCL + 3°) were compared. These landmarks are able to be accurately compared by placing a sizing guide on the distal cut surface of the femur and dialling the rotation between the two sets of pin-holes. Therefore, the rotational angle between the PCA and SL was able to be accurately determined. When they were different, an average position was produced as accurately as possible by altering the rotation of the sizing guide in 1° increments. This average position was marked with a further set of pin-holes, and the 4-in-1 cutting block was inserted into these pin-holes. The prosthesis was therefore inserted in the average position. Therefore, the relationship between the PCA or SL and the actual component position was able to be determined from the photograph. An intraoperative photograph was taken of the three sets of pin-holes using a camera set in the overhead light (Fig. 3).

The procedure was completed using one of two types of uncemented femoral components (Triathlon CR, Stryker Co., Kalamazoo MI, or Active, Allegra Orthopaedics, Sydney, NSW, Australia). Both components have symmetrical posterior condyles.

A postoperative computed tomography (CT) scan was performed prior to discharge from hospital. A low-dose scanner (GE Optima 660) produced 1.25 mm slices from the proximal end of the femoral component to the tip of the tibial stem.

Measurements were taken of both the intraoperative photographs and the CT scan by two observers (one orthopaedic resident and one orthopaedic surgeon). The first ten cases were measured twice at an interval of 1 week to assess interobserver and intraobserver reliability. Internal rotation was recorded as a negative angle and external rotation as positive. The angles between the three sets of pinholes were measured on the photographs using Adobe Photoshop CS6. The angle between the posterior condyles of the femoral component and the SEA was measured using InteleViewer 4-3-4. The SEA was defined as a line from the most prominent point on the lateral epicondyle to the deepest point of the medial sulcus posterior to the medial epicondyle. Multiple axial slices were referenced when necessary to determine the landmarks. If either observer found that the sulcus was not able to be accurately determined, the scan was excluded. The data was collected using a purpose-designed Excel spreadsheet. Statistical analysis was performed using SPSS (22.0.0.0).

The postoperative component position (“actual component position”) (Table 1) was measured on the CT scan relative to the SEA. On the intraoperative photograph, the position in which the component was inserted was determined relative to the SL and PCA. By combining these measurements, the positions of the PCA, PCL, and SL relative to the SEA were calculated. The position which would have been achieved if the PCA and SL could be perfectly averaged intraoperatively (*calculated mean PCA and SL to SEA*) (Table 1) was also calculated.

**Table 1:** Rotational measurements

	Mean	SD	Range
SL to SEA	−0.7°	2.3°	−5.5° to +4.6°*
PCL to SEA	−2.1°	1.9°	−9.1° to +2.0°*
PCA to SEA	+0.9°	1.9°	−6.1° to +5.0°
PCL to SL	−1.4°	3.2°	−10.6° to +6.3°
PCA to SL	+1 6	3.2°	−7.6° to +9.3°
Actual component position	+0.6°	1.4°	−3.8° to +4.0°**
Calculated mean PCA and SL to SEA	+0.1°	1.4°	−3.7° to +2.7°*

Positive measurements are externally rotated

*Decreased variance of *calculated mean PCA and SL to SEA* when compared to either *SL* (*F* = 15.805, *p* < 0.001) or *PCA* (*F* = 7.068, *p* < 0.001) individually

**Decreased variance of *actual component position* when compared to either *SL* (*F* = 22.634, *p* < 0.001) or *PCA* (*F* = 4.902, *p* < 0.05) individually

A definition of trochlear condylar divergence (TCD) was developed to identify cases in which the PCL and SL were not perpendicular. This is a measure of femoral rotational asymmetry. A difference of 4° was deemed clinically significant based on the evidence that more than 3° of femoral malrotation is associated with adverse patellofemoral outcomes [2, 3, 6].

Subgroup analysis was performed based on the predominant pattern of arthritis detected on preoperative knee X-rays and intraoperative findings. They were classified as predominantly medial, lateral, patellofemoral or tricompartmental osteoarthritis.

## Results

Of the 91 cases, one was excluded as the SL could not be clearly delineated. This patient had a patellectomy 30 years prior to TKA and the groove was grossly deformed. Six cases were excluded due to a poorly visible medial epicondylar sulci on the postoperative CT scans, leaving 84 cases for analysis. Slightly, more female (44, 52%) than male (40, 48%) participants were included in the study. More TKAs were conducted on the right side (46, 55%).

The SL 0.7° internally rotated to the SEA (SD 2.3°, range −5.5° to +4.6°). The PCL was 2.1° internally rotated (SD 1.9°, range −9.1° to +2.0°). The actual component position achieved was 0.6° externally rotated (SD 1.4°, range −3.8° to +4.0°). The calculated mean PCA and SL was 0.1° externally rotated (SD 1.4°, range −3.7° to +2.7°). There was a significant decrease in variance between the calculated mean PCA and SL to SEA to either the *SL* (*F* = 15.805, *p* < 0.001) or the *PCA* (*F* = 7.068, *p* < 0.001) (Table 1). There was also a significant decrease in the variance between the a*ctual component position* to either the SL (*F* = 22.634, *p* < 0.001) or the PCA (*F* = 4.902, *p* < 0.05).

There was a significant difference between the means for both the SL and PCA when compared to either the actual component position or the calculated mean PCA and SL to SEA (*p* < 0.05).

There was a significant difference between the means for the SL, PCA and actual component position compared to the SEA (*p* < 0.05). There was no significant difference between the SEA and the calculated mean PCA and SL to SEA (*p* > 0.05).

Subgroup analysis did not find any difference in the means or variability of the SL or PCA measurements between patients with predominantly medial, lateral or patellofemoral osteoarthritis (Table 2).

**Table 2:** Subgroup analysis

	Medial	Lateral	Patellofemoral	Tricompartmental
SL to SEA	−0.8° ± 2.2°	−0.7° ± 3.1°	−0.1° ± 1.1°	−0.5° ± 2.8°
PCA to SEA	1.1° ± 1.9°	0.2° ± 1.8°	0.1° ± 1.3°	1.3° ± 1.8°
Calculated mean PCA and SL to SEA	0.1° ± 1.4°	−0.2° ± 1.3°	0.0° ± 1.0°	0.4° ± 1.3°
PCA to SL	1.8° ± 3.0°	0.8° ± 4.4°	0.3° ± 1.4°	1.8° ± 3.8°

Comparative analysis found no significant difference in means or variance amongst any of the subgroups (all *p* > 0.05)

Outliers were considered to be measurements more than 3° from the SEA. By combining the SL and PCA, the rate of outliers decreased from 19% for the SL, 17% for the PCA to 2% for the calculated mean PCA and SL (*p* < 0.05) (Table 3) and 2% for the actual component position (*p* < 0.05).

**Table 3:** Outliers^a^ and Trochlear condylar divergence (TCD)^b^

	Medial (*n* = 59)		Lateral (*n* = 11)		Patellofemoral (*n* = 5)		Tricompartmental (*n* = 9)		Total (*n* = 84)	
	*n*	%	*n*	%	*n*	%	*n*	%	*n*	%
SL	9	15	4	36	0	0	3	33	16	19*
PCA	10	17	2	18	0	0	2	22	14	17*
Combined PCA and SL	2	3	0	0	0	0	0	0	2	2*
Trochlear condylar divergence (TCD)	14	24	4	36	1	20	3	33	21	25

^a^ *Outliers* defined as more than 3° internally or externally rotated to SEA

^b^ *Trochlear condylar divergence* defined as difference between SL and PCL >4°

*Combined PCA and SL vs *SL* (*p* < 0.05); *combined PCA and SL* vs *PCA* (*p* < 0.05)

The rate of femoral rotational asymmetry or trochlear condylar divergence was <25% (21/84).

Interobserver and intraobserver reliability was excellent for all measurements (all Pearson’s coefficient >0.95).

## Discussion

The STAG device removes parallax errors which occur when referencing APA [26]. These errors occur because the coronal alignment of the trochlear groove is highly variable [18, 26]. Feinstein et al. [29] also reported that the coronal alignment of the groove was highly variable with a range from 6.7° valgus to 7.7° varus to the mechanical axis. These results have been reproduced using 3DCT scan techniques [18, 26]. This coronal variation needs to be considered when measuring the rotational alignment of the groove. By correcting for this variation and looking directly along the coronal direction of the trochlear groove in each individual, the trochlear groove becomes a much more reliable landmark than Whiteside’s line. This results in a more accurate landmark which reflects the true rotational alignment of the trochlear groove and which more closely parallels the SEA [26]. This can be achieved by preoperative planning using CT scans, and correcting for the coronal alignment, or intraoperatively with the STAG device. Currently, there are no computer navigation systems which allow for this error to be corrected.

The combination of the SL and PCA produced fewer outliers than predicted by the findings of the 3DCT study suggesting that the intraoperative technique using the STAG is at least as accurate and reproducible as the virtual technique using the CT scans.

On average, the SL was slightly internally rotated to the PCA and SEA, and externally rotated to the PCL. Referencing the SL using the STAG device produced a landmark with a narrow range and a level of variability which was similar to the PCA. This is an improvement compared with published results using APA [30–32] which have indicated a wide range and high degree of variability.

**Fig. 4:**
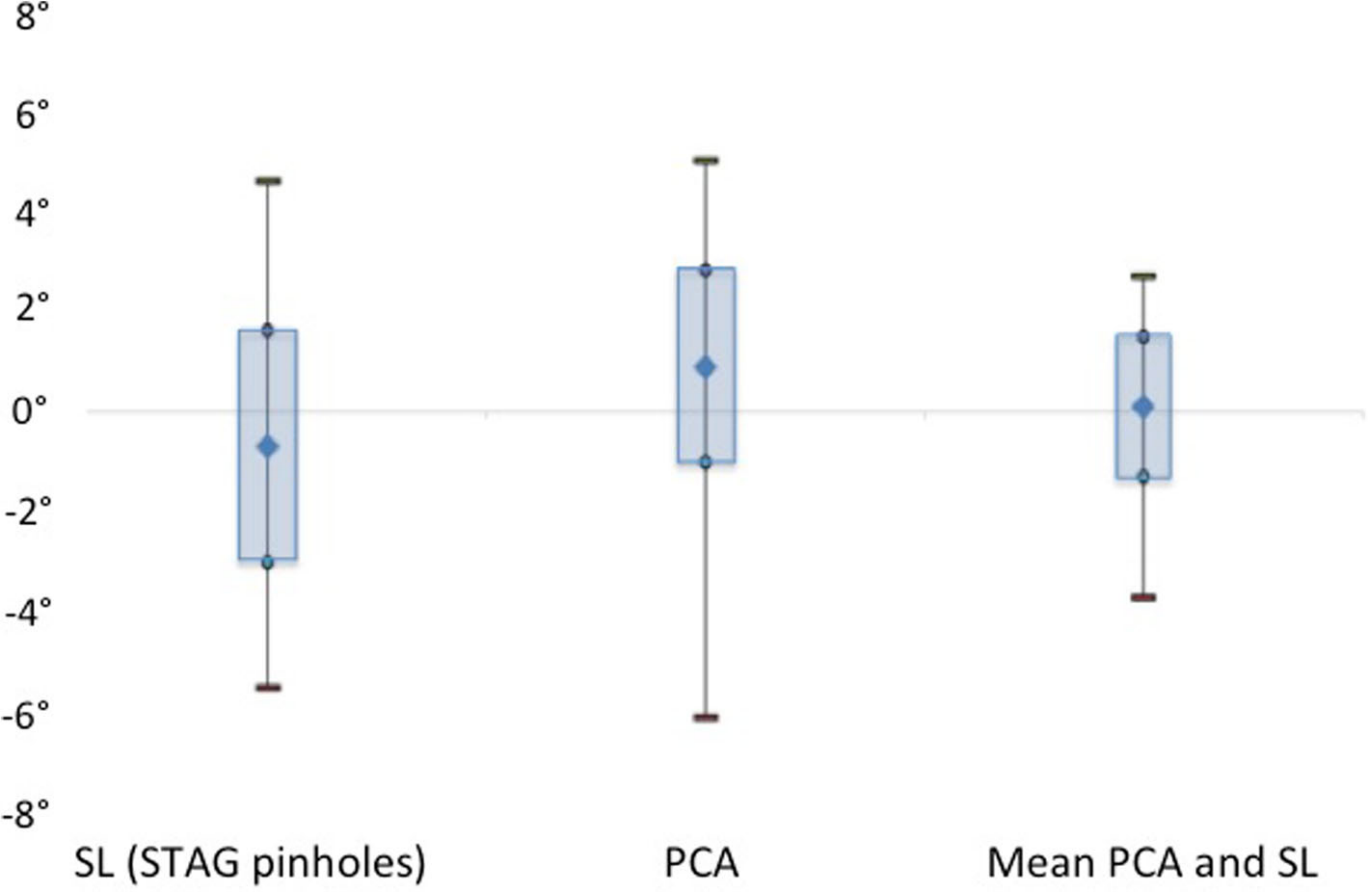
Averaging the SL and the PCA reduces the variability of component positioning relative to the SEA

The comparison of the means revealed that the only technique to produce no statistically significant difference from the SEA was the calculated mean PCA and SL to SEA. While this would support the hypothesis that combining the two landmarks is likely to result in a closer average position relative to the SEA, it is the decrease in variability and outliers which is more important. In addition, the size of the difference in the means relative to the SEA was less than 1° for each of the techniques, which may not be clinically significant. It is the size of the potential variation using each technique which is important. This changes from up to 5.5° for the SL and 6.1° for the PCA down to 4.0° for the actual component position and 3.7° for the calculated mean PCA and SL to SEA. Averaging the SL and PCA (Fig. 4) produced a significant decrease in both the overall variability and the number of individual outliers. Indeed, the outlier rate achieved was reduced from 18% using PCA alone, or 19% using SL alone, to 2% by combining the landmarks. This compares very favourably with results using other techniques including gap-balancing [33] and measured resection [32, 34–36].

Subgroup analysis showed that the results were equally effective for medial, lateral, tricompartmental and patellofemoral osteoarthritis. The SL was very accurate in cases of predominantly patellofemoral osteoarthritis (mean −0.1°, SD 1.1°). When severe trochlear arthritis is present, there are often parallel grooves in the bone surface which allow easy identification of the SL. In knees with lateral arthritis, there was on average 0.9° of internal rotation of the PCA compared to the medial group. This difference was not significant. There was no difference in the SL between medial and lateral groups.

**Fig. 5:**
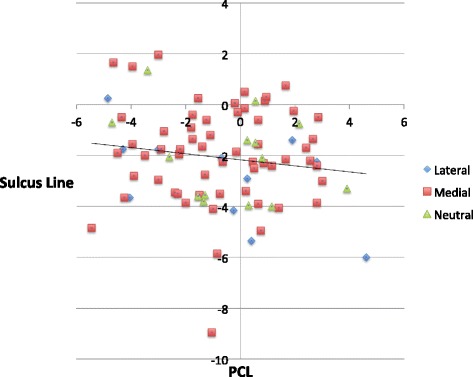
Graph of data showing the high degree of asymmetry between the posterior condyles and the trochlear groove. The direction of asymmetry is not related to the posterior condylar wear from the medial or lateral location of the arthritis

Analysis of the relationship between the SL and PCL revealed a high percentage of cases in which the two landmarks were not perpendicular to each other (mean difference 1.4°, SD 3.2°, range −10.6° to 6.3°). Overall, there was only a weak negative correlation between the SL and PCL (Fig. 5). The relationship between the SL and PCL is not predictable and is not correlated with the pattern of arthritis (Table 3).

In 21 of 84 knees, there was more than 4° of difference between the PCL and SL. We have coined the term trochlear condylar divergence to describe this group. In 19 of these 21 knees, the SL shifted, relative to the SEA, in the opposite direction to the PCL. This suggests an anatomical variation in which the rotational alignment of the SL and the PCL may be linked. The direction of this divergence was not influenced by the diagnosis of medial or lateral osteoarthritis. In two of four knees with lateral arthritis and nine of 14 knees with medial arthritis, the divergence occurred in the opposite direction to that anticipated if the variation was due to posterior condylar bone loss. For example, in nine knees with medial arthritis, the PCL was more than 4° internally rotated to the SL. This is despite posterior condylar cartilage and bone loss tending to externally rotate the PCL. Therefore, if the surgeon in these nine cases had internally rotated the femoral component relative to the PCA in order to compensate for posterior medial condylar bone loss the degree of internal malrotation relative to the SEA and SL would have increased. This would indicate that it is a true anatomical variation rather than the effect of arthritic bone loss. Further studies are planned to assess this variation and determine if it is linked to proximal tibial coronal alignment.

These findings are consistent with the findings of Iranpour et al. [18]. This study used a different technique for measuring the trochlear groove. It also plotted multiple points along the floor of the groove and noted the variation in coronal alignment of the groove. However, they did not remove the parallax error by compensating for this coronal variation. The rotation of the posterior condyles was derived using a sphere-matching technique. The line of the trochlear groove was determined to be “close to parallel” to the transcondylar axis with an average of 1° of external rotation. However, there was a very large degree of variability between the two landmarks, with a standard deviation of 3°. Some of this variability will be due to parallax error, and the remainder will be due to anatomical variation.

Iranpour et al. [19] also published a cadaver study in 2010 which measured variation between the posterior condyles and the path of the patella during flexion. They found that the path of the patella was 88.8° ± 3.8° from their condylar axis. This means that it was on average 1.2° externally rotated to the condyles. This is similar to our finding of the SL being 1.4° externally rotated to the posterior condyles with a SD of 3.2°.

Both studies by Iranpour and colleagues are quoted by proponents of posterior referencing and kinematic alignment as evidence of a consistent rotational relationship between the posterior condyles and the patellofemoral joint [37]. This conclusion ignores the large degree of variability between the landmarks which is reinforced by our findings. A more appropriate conclusion is that femoral axial anatomy is frequently asymmetrical with divergent rotational alignment between the posterior condyles and the patellofemoral joint.

This is also consistent with several recent studies which measured the relationship between the posterior condyles, the epicondyles and the trochlear groove using a variety of techniques. Jones et al. [38] used MRI scans and found that “only 24% had an external rotation angle between 2.5° and 3.5° relative to” the posterior condyles. Loures et al. [24] measured a range of 11.75° for the PCL and 14.29° for the APA on MRI scans. Theinpont et al. [20] looked at 2637 3D reconstructions of preoperative CT scans and predicted a “41% risk of malalignment” from referencing the posterior condyles. Cinotti et al. [39] found that combining the posterior condyles, epicondyles and anteroposterior axis using computer navigation reduced the need for lateral retinacular release over referencing the posterior condyles alone. Several studies have recommended combining the anterior and posterior landmarks [24–26, 39, 40].

The results of this study raise concerns regarding any technique which solely references the posterior condyles to determine rotation. Twenty-five percent of femora have more than 4° of rotational asymmetry, and yet our femoral components are symmetrical. Therefore, referencing solely the posterior condyles may produce a well-balanced tibiofemoral joint but change the rotation of the patellofemoral joint relative to its original position. This may be particularly pertinent in cases of kinematic alignment in which the native knee has a combination of proximal tibial varus and external rotation of the SL relative to the PCL. This combination may lead to an exaggerated internal rotation of the trochlear groove of the femoral component relative to the native position.

One limitation of this study is that we did not attempt to compensate for posterior condylar bone loss when determining the PCA. This was done to maintain a consistent technique and to avoid subjectivity involved in compensating for bone loss intraoperatively. Nam et al. [41] determined that posterior condylar bone loss is a very late event in the arthritic process and that the degree of correction required is small. In addition, our subgroup analysis of outliers demonstrated that compensating for femoral condylar bone loss would have increased the degree of malrotation in 65% of varus knees and 50% of valgus knees. There are likely to be cases with severe posterior condylar bone erosion in which this should be taken into account prior to referencing the posterior condyles, however, relying solely on the posterior condyles to determine femoral component rotation will result in an unacceptable rate of malrotation. Further to this, it is clear that correcting for bone loss would not improve the consistency of the relationship between the PCL and SL. There clearly will be cases in which either the posterior condyles or trochlear groove are grossly deformed and should not be referenced. However, these cases are rare and in general the concept of averaging landmarks is valid for the vast majority of knees.

Intraoperatively, the ability to perfectly average the SL and PCA was limited by the sizing guide which had 1° or 1.5° rotational increments. In cases where the difference between the SL and PCA was less than the 1° the guide was left at the PCA landmark. Therefore, a measurement based on perfect averaging of the angles from the intraoperative photographs was also calculated. This is why there are "actual" and "calculated" measurements for the average of the SL and PCA. The difference between the two results was minor.

Further limitations of the study involve the exclusion of several cases due to difficulty finding the medial epicondylar sulcus; however, our exclusion rate is similar to other studies [2, 22].

Due to the lack of preoperative long leg X-rays, data on mechanical alignment or severity of deformity was not available. Preoperative knee X-rays and intraoperative assessment of the location of the arthritis were used to produce subgroup data. There are only 11 cases with predominantly lateral compartment osteoarthritis. The results in this group suggest that combining the two landmarks may be a reliable technique; however, the numbers are too small to draw firm conclusions. Further studies assessing non-arthritic knees are planned.

Measurement errors could occur with the identification of the SL and measurement of the intraoperative photographs and the CT scans. However, the very high interobserver and intraobserver reliability makes it unlikely to account for the degree of variation we have identified. Likewise, the measurement error associated with the use of the STAG device was shown to be small (mean 1° for both interobserver and intraobserver measurements) in the previously published cadaver study [25].

## Conclusions

A high proportion of femora have axial asymmetry with different rotational alignment of the trochlear groove and posterior condyles. By using a trochlear alignment guide that corrects for the coronal alignment of the trochlear, the rotational alignment of the groove can be more accurately identified during surgery than by using APA. It also allows direct comparison between the anterior and posterior landmarks. Averaging the SL of the trochlear groove and the PCA significantly decreases femoral component malrotation.

## Abbreviations

3DCT: Three-dimensional computed tomography
AEA: Anatomical epicondylar axis
APA: Anterior posterior axis
CT: Computed tomography
FEA: Flexion-extension axis
IM: Intramedullary
KA: Kinematic alignment
MRI: Magnetic resonance imaging
PCA: Posterior condylar axis
PCL: Posterior condylar line
SEA: Surgical epicondylar axis
SL: Sulcus line of the trochlear groove
STAG: Sulcus line Trochlear Alignment Guide
TCD: Trochlear condylar divergence
TKA: Total knee arthroplasty
